# Risk indexation and atrial fibrillation

**DOI:** 10.18632/aging.101889

**Published:** 2019-03-28

**Authors:** Thanh H. Nguyen, Tamila Heresztyn, John D. Horowitz

**Affiliations:** 1 The Queen Elizabeth Hospital, Department of Cardiology, University of Adelaide, Woodville South, Australia; 2 Basil Hetzel Institute, Woodville South, Australia

**Keywords:** asymmetric dimethylarginine (ADMA), symmetric dimethylarginine (SDMA), atrial fibrillation, thromboembolism

The prevalence of atrial fibrillation (AF) increases markedly with patient age [1], for reasons which have never been fully understood in the past. Occurrence of AF represents a basis for haemodynamic deterioration, due to inadequacy of diastolic filling of the left ventricle, and also impairment of the ventricular force-frequency relationship in patients with concomitant valvular or hypertensive heart disease. Furthermore, AF is a major risk factor for intra-atrial thrombosis and resultant thromboembolism. Overall, AF represents a basis for markedly increased risk of disability and death.

While optimisation of haemodynamic status in patients with AF represents a basis for ongoing clinical difficulty in many cases, there is now general agreement that most patients benefit from long-term anticoagulation, with the recent development of direct oral anticoagulants (DOACs) increasing the safety profile of this strategy beyond that seen with warfarin. In particular, the risk of intracranial bleeding is markedly diminished with DOAC therapy.

It is also appropriate for there to be improved understanding of the mechanisms underlying the development of AF and the associated thromboembolic risk. AF has always been considered to represent a disorder resulting from atrial distension alone, but on the other hand some cases occur in the absence of haemodynamic perturbation and/or abnormal hormonal milieu, suggesting that abnormal myocardial biochemical processes may underlie the emergence of AF. Indeed, there is increasingly strong evidence that a combination of inflammatory activation and impaired nitric oxide (NO) generation and signalling may account for predisposition to AF. Specifically, activation of the neutrophil enzyme myeloperoxidase (MPO) has been shown to be a pivotal biochemical factor [2]. As regards NO, there is abundant evidence of an association between endothelial dysfunction and AF [3] and it has been shown that NO signalling in circulating platelets is markedly impaired in new onset AF [4]. Furthermore, normal ageing is associated with progressive attenuation of NO signalling [5], coupled with increased tissue expression of pro-inflammatory thioredoxin-interacting protein (TXNIP). Hence there should be little surprise that AF tends to occur in most frequently in ageing patients with demographics favouring the occurrence of endothelial dysfunction.

What about the risk of thromboembolism in patients with AF? In the past, it has been assumed that this risk was governed primarily by Virchow’s triad [6]: indeed empirically derived indices of thromboembolic risk such as CHADS_2_ and CHA_2_DS_2_-VASc include heart failure as a predictor. However, many of the parameters included in these indices are also closely associated with endothelial dysfunction and/or inflammatory activation. Therefore, as with the occurrence of AF, there is a strong reason to seek the biochemical bases for thromboembolic risk.

Recently published data [7] have been based on analyses of plasma concentrations of the methylated arginine concentration derivatives asymmetric (ADMA) and symmetric (SDMA) dimethyl arginine in 5004 patients participating in the ARISTOTLE trial, which compared warfarin with the DOAC Factor Xa inhibitor apixaban in patients with AF. The objective of this prospectively planned substudy was to ascertain whether ADMA, which functions as a competitive inhibitor of nitric oxide synthase, and SDMA, which is largely an inflammatory activator, might be mediators of risk of outcomes (thromboembolism, major bleeding and death) in these patients, and whether ADMA and/or SDMA accumulation might account for individual risk scores. It was found that ADMA and SDMA concentrations correlated directly with markers of both thromboembolic and bleeding risk. Furthermore, after multivariable analyses, ADMA concentrations were independently (although weakly) predictive of thromboembolic risk, and strongly of bleeding and mortality risk. SDMA concentrations were very strongly predictive of risk of bleeding and of mortality.

**Figure 1:**
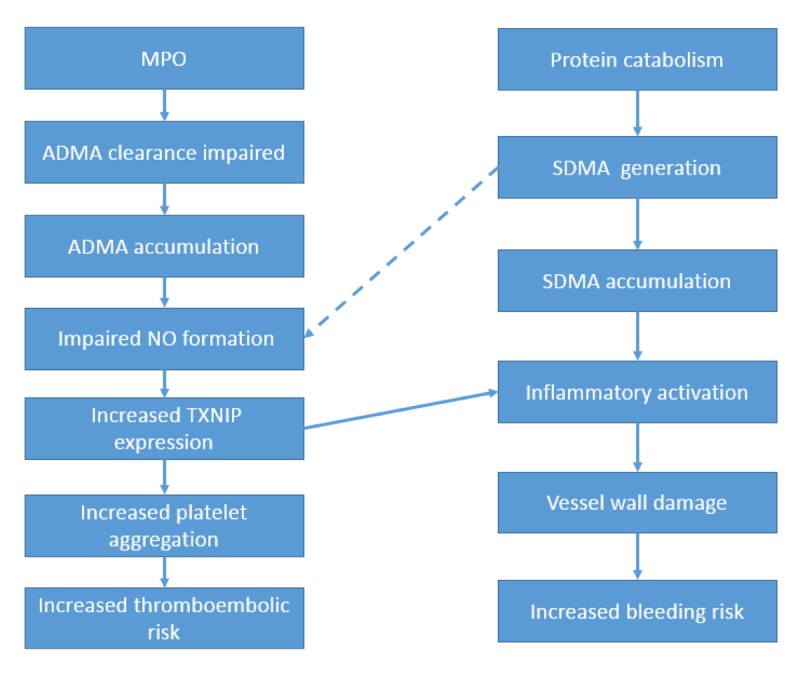
**Schematic.** Postulated biochemical bases for observed nexus between elevated plasma concentrations of ADMA/SDMA and outcomes in AF patients.

It therefore appears that, among anticoagulated patients with AF, markers of both impaired NO effect and of inflammatory activation serve as biochemical markers of risk of major adverse events in patients with AF, and this utility is incremental to that of the usual clinical parameters. It must be emphasised that the impact of ADMA accumulation may in part reflect actions of MPO, as MPO inhibits the metabolic clearance of ADMA [8]. Potential biochemical modulation of the current findings is summarized in Figure 1.

What are the practical implications of these findings? First, it is likely that they explain, at least in part, the substantially increased risk of bleeding complications in anticoagulated patients with AF and renal insufficiency, since both ADMA and SDMA have substantial components of renal clearance. Indeed, the incremental impact of measuring ADMA and SDMA concentrations in predicting major bleeding is so marked that it may serve as a means for decision-making before anticoagulation is undertaken in frail elderly individuals. Finally, the current results point to the potential clinical utility of developing treatments capable of lowering plasma ADMA/SDMA concentrations.
